# Homelessness among psychiatric inpatients in North Rhine-Westphalia: a retrospective routine data analysis

**DOI:** 10.1186/s12888-022-03786-6

**Published:** 2022-02-19

**Authors:** Ida Sibylle Haussleiter, Isabell Lehmann, Bianca Ueberberg, Josephine Heinz, Jürgen Zielasek, Euphrosyne Gouzoulis-Mayfrank, Georg Juckel

**Affiliations:** 1 LWL Research Institute for Mental Health, Ruhr University Bochum, LWL University Hospital, Bochum, Germany; 2 Department of Psychiatry, Ruhr University Bochum, LWL University Hospital, Alexandrinenstrasse 1, 44791 Bochum, Germany; 3 LVR Institute for Healthcare Research, LVR Institute for Research and Education, Wilhelm-Griesinger-Strasse 23, 51109 Cologne (Köln), Germany; 4 Medical Faculty, Heinrich Heine University, Düsseldorf, Germany; 5 LVR Clinics Cologne, Cologne, Germany

**Keywords:** Homelessness, Mental healthcare, Mental disorders, Hospital data, Routine data analysis

## Abstract

**Background:**

Within the last five years the number of homeless persons in Germany has more than doubled, with many suffering from mental illnesses that require treatment. Whether the mental illness itself led to losing shelter or whether the state of being homeless increased the likelihood of developing symptoms of a mental disorder remains unclear. The current study assessed the interaction of homelessness and mental illness from a care provider perspective.

**Methods:**

We conducted a retrospective analysis of inpatient routine data from 20 psychiatric hospitals in North Rhine-Westphalia (NRW), Germany, over a period of four years (*N* = 366,767 inpatient treatment cases). Patients were considered “homeless” if they had no fixed unique address.

**Results:**

About 2.4% of the analyzed cohort was classified as homeless, with increasing tendency over the study period (+14% from 2016 to 2019). The percentage of homeless patients varied broadly between the hospitals (0.2–6.3%). Homeless patients were more often male and on average eight years younger than patients with a fixed address. Homeless patients experienced more involuntary measures (admission and restraint), had a shorter course of treatment and were more often discharged within one day. Every second homeless case was diagnosed with a substance use disorder and every third homeless case with a psychotic disorder, whereas affective disorders were diagnosed less frequently in this group. Psychiatric comorbidity occurred more often in homeless patients whereas somatic diseases did not.

**Conclusions:**

Multiple patient-related sociodemographic and local factors are associated with homelessness of psychiatric inpatients. In addition, clinical factors differ between homeless and non-homeless patients, pointing to more severe mental illness and treatment complications (e.g., coercive measures) in homeless persons. Thus, homelessness of psychiatric inpatients can imply special challenges that need to be considered by healthcare providers and politicians, with the goal of optimizing mental and social care and the mental health outcomes of homeless persons.

## Background

Homelessness is considered an increasing social problem in developed countries [20]. The overall mortality and morbidity, including mental health problems, are clearly higher among homeless persons than amongst the general population [2, 18]. Mental disorders are also among the risk factors for age-standardized excess mortality rates in this group [29], which are 2–5 times higher than in the general population. Since the burden of psychiatric morbidity in homeless persons is substantial and there are strong indications of a complex relationship between homelessness and mental disorders as they promote each other and lead to poor healthcare outcomes [3, 18].

In Germany, there were approximately 678,000 homeless persons in 2018, a figure that had doubled within four years (2014–2018) mainly due to the large numbers of refugees in that period [5]. Within the last 20 years, there have been some studies on the mental health situation of homeless persons in different German cities. The most recent of these local studies evaluated 232 randomly drawn homeless people in the Bavarian metropolitan city of Munich during 2010–2015 and reported that psychiatric treatment started on average of six years before the persons became homeless [3]. A recent meta-analysis of 11 German studies with an overall study population of 1220 homeless persons in Germany resulted in 77.4% clinical disorders [according to the Diagnostic and Statistical Manual of Mental Disorders, Fourth Edition (DSM-IV), axis I, pooled point prevalence of eight studies], primarily substance use disorders (SUD) [38]. These data thus yielded a mental illness rate 3.8 times higher than in the general population [21]. The gender ratio in this largest German meta-analysis on mental health in homeless people was 1:9 in favor of the male gender [38].

Another perspective on the problem of homelessness and mental disorders offers the analysis of homelessness in persons who receive mental hospital care. The prevalence of homelessness among psychiatric inpatients varies considerably between studies from 0.55-39% [30, 44]. The most recent German study on the housing situation of psychiatric inpatients was the WOHIN- study. This cross-sectional patient survey included 540 inpatients in Berlin with a defined catchment area in an underprivileged district with approximately 270,000 inhabitants [40]. Due to the study design, 43.2% of all admitted patients in the study period were included into the survey and out of those, only 68.7% of the patients lived in an own apartment, whereas 13.0% were homeless and 18.3% were accommodated in sociotherapeutic facilities. Another German Study [45] included all 3174 admissions within a 12months period and observed homelessness in 31% of the patients. This relatively high percentage results from homeless definition as “no private home” and therefore count of all patients who lived in sociotherapeutic facilities. Another European study from Switzerland analyzed the data on 16247 patients consecutively referred to psychiatric hospitals over a four-year time period and observed a rate of 1.60% homelessness among them. Unlike traditional homelessness interventions with prerequisites for treatment or sobriety, recovery-oriented interventions such as Housing First in the USA and At Home/Chez Soi in Canada [35] offer permanent housing to participants immediately [42] and have been shown to reduce use of several types of health and social services among homeless individuals with mental illness [7]. User experiences with Housing First compared to treatment-first approaches have been published in North American context, but less is known for European context. A recent study compared experiences in seven European countries [16], but so far there are no comparable German studies on such low-demand supportive housing. Our study “Homelessness in persons with mental illness – risk factors, impact and interventions: An overview in North Rhine-Westphalia” (WohnLos) aimed at identifying the magnitude of the problem of homelessness in mentally ill persons in North Rhine-Westphalia (NRW) and at identifying the risk profiles of persons affected by homelessness. In the present retrospective part of our study, we analyzed routine data from a large sample of inpatients from 20 mental hospitals regarding the prevalence of homelessness. The hospitals are spread over the federal state of NRW, the fourth largest state in Germany by area and the largest state by population size (17.9 million inhabitants). The hospitals have a catchment area for mental healthcare for about half of the population of NRW. In 2020, the state of NRW counted 49,987 homeless persons [26], which is about 0.28% of the NRW population. A nationwide survey is scheduled for 2022. Our study aimed to assess the prevalence of homelessness among psychiatric inpatients in different areas of NRW and to identify the risk factors for being homeless at time of admission. In addition, our study aimed to analyze the routine care provided to homeless people in psychiatric hospitals compared to non-homeless patients. Due to our high patient case number and the large geographic area analyzed, our routine data analysis may provide more representative data than former studies.

## Methods

### Setting

Data were collected from 20 psychiatric hospitals belonging to the two communal councils of the federal state of NRW: the Council of Westphalia [Landschaftsverband Westfalen-Lippe (LWL), with eleven hospitals] and the Council of Northrhine [Landschaftsverband Rheinland (LVR), with nine hospitals]. Together, the LWL and LVR hospital groups form the biggest providers of psychiatric services in Germany, covering both rural and urban areas (6969 hospital beds). Each of the eleven LWL and nine LVR hospitals has a defined catchment area. The number of inhabitants at the hospital sites was 260,141 on average (range 12,933–1,087,863) or 1321 per km^2^ (range 107–2860). According to the DeStatis [9] definition, most of the sites were classified as urban areas, four hospitals were located in semi-urban areas and no hospital was located in an exclusively rural area. The annual point-in-time count in 2019 registered an average of 1712 homeless persons (range 277–6198) at the hospital sites, corresponding to a rate of 1368 per 100,000 persons [26].

### Procedures

A retrospective, large-scale multicenter comparative study of psychiatric treatment cases was carried out in the 20 LVR and LWL hospitals for the years 2016–2019. We analyzed hospital reimbursement data and additional routine data from the medical records of each case and combined these data with data on socioeconomic environments, such as urbanization. The hospital reimbursement data (Section 301 of Social Code Book V) are transferred to health insurance companies as part of the daily routine, thus leading to the accumulation of a reliable and comprehensive database of service utilization data for all patients treated in the LVR and LWL hospitals. Diagnoses according to the Tenth Revision of the International Classification of Diseases (ICD-10) were based on discharge records and up to 20 diagnoses were considered. The first two digits of the psychiatric main discharge diagnosis encoded in the system were used to specify the diagnostic subgroups of each case (such as ICD-10 code F2 for the group of psychotic disorders).

The housing status was categorized based on the documented housing situation at time of admission. For each hospital, we requested the common documentations for “homelessness”. These included: “without permanent address” in the patients’ address line; specific addresses, such as the address of the local train station or local homeless shelters; or the ZIP code “9999”. Patients were subdivided into two groups corresponding to their housing situation (homeless vs. non-homeless). “Homeless” meant the absence of a fixed, unique address as defined by the European Commission [12]. This definition comprises the first two categories of the existing European Typology of Homelessness and Housing Exclusion (ETHOS) [11]: roofless (sleeping rough, unsheltered) and houseless (living temporarily with other persons or in transitional accommodation).

Pseudonymized data were provided by the IT department of the LWL and the LVR statistics database. All inpatient data were considered; 367,551 cases were registered in this period, of which 366,767 treatment cases were analyzed after data cleaning according to minimum data criteria (age, gender, main diagnosis). Readmissions were considered as new treatment cases in this design. The socioeconomic data at the hospital sites were obtained from official sources/databases such as Statistisches Bundesamt (destatis.de), Sozialberichterstattung NRW, Ministry of Work, Health and Social Matters of the federal state of NRW (Ministerium für Arbeit, Gesundheit und Soziales: MAGS) and from publications of the corresponding cities and communities (wegweiser-kommune.de). The study was approved by the responsible ethics committees for the LWL and LVR and all medical hospital directors gave their written consent.

### Data analysis

Data were collected retrospectively. Analyses were carried out using a statistical software package (IBM SPSS Statistics 26.0^®^). Group differences (homeless vs. non-homeless cases) were analyzed using Pearson’s chi-square test and/or Fisher’s exact test for categorical variables and two-tailed *t*-tests for independent variables. A *p* value of less than 0.05 was interpreted as statistically significant. The main diagnosis was encoded dichotomously (F2 yes/no) and statistical testing was performed for each diagnosis separately. The housing status was also dichotomous (homeless or non-homeless). Principal component analysis (PCA) and binary logistic regression were performed.

## Results

A total of 366,767 treatment cases (57.3% male, mean age 47.52 ± 18.31 years) were analyzed, of which 8636 cases (2.4%) were classified as homeless. The number of annual treatment cases decreased within the study period, from 92,837 in 2016 to 89,765 in 2019. Cases classified as homeless increased from 1998 to 2284 cases per year (+14%) in the same period, whereas the percentage of homeless patients increased only slightly (2016: 2.15%; 2017: 2.37%; 2018: 2.36%; 2019: 2.54%). Compared to the average prevalence of homelessness among the population at the hospital sites [26], the percentage among the inpatients of the psychiatric hospitals is higher. When looking at the different hospitals, the rate of homelessness among patients varied from 0.2% to 6.3%. The highest percentages were found in hospitals located in large and medium-size cities (Cologne: 6.3%,Münster: 5.8%; Düsseldorf: 3.7%; Paderborn: 3.2%; Essen: 3.1%) and the lowest percentages were found in small towns (Hemer: 0.2%; Gütersloh: 0.2%; Marsberg: 0.5%; Langenfeld: 0.9%).

Table 1 displays the demographic/clinical characteristics and group differences for the total cohort and Table 2 displays the results regarding hospital treatment. With regard to gender, 56.8% of the non-homeless and 80.2% of the homeless patients were male (*p* < .001). The two groups (homeless vs. non-homeless) differed significantly in age (*p* < .001), with the homeless patients being, on average, eight years younger. The three most frequent main diagnostic clusters were substance use (ICD-10 diagnostic code F1), psychotic disorders (F2) and affective disorders (F3). Substance use (F1), psychotic disorders (F2) and personality disorders (F6) occurred more frequently within the homeless subgroup, with affective disorders (F3) diagnosed less frequently in this group. Every second homeless case was diagnosed with a SUD(F1: 50.7%) and every third case was diagnosed with a psychotic disorder (F2: 30.0%). Neurotic, stress-related and somatoform disorders did not differ between homeless and non-homeless patients but the overall occurrence was low, with less than 5% in all cases. In the homeless group, somatic comorbidity was less frequently diagnosed, whereas psychiatric comorbidity occurred more frequently. We tested two double diagnoses (F1+F2 and F1+F6) in relation to being homeless versus not being homeless and found significant associations between both double diagnoses and homelessness.

**Table 1:** Demographic/clinical characteristics and group differences

	**Total** **(*N* = 366,767)**	**Non-homeless** **(*n* = 358,131)**	**Homeless** **(*n* = 8636)**	**Group comparison**	***p***
Demographic characteristics					
Male gender (*n*, %)	210,228 (57.3)	203,306 (56.8)	6922 (80.2)	χ^2^_(1)_ = 1884.85	< .001
Age (years; mean, SD)	47.52 (18.31)	47.72 (18.39)	39.27 (12.3)	*t*_(9589.19)_ = 62.15	< .001
Clinical characteristics (main discharge diagnosis yes/no)					
F0 (*n*, %)	33,860 (9.2)	33,768 (9.4)	92 (1.1)	χ^2^_(1)_ = 703.92	< .001
F1 (*n*, %)	126,221 (34.4)	121,844 (34.0)	4377 (50.7)	χ^2^_(1)_ = 1037.09	< .001
F2 (*n*, %)	64,414 (17.6)	61,820 (17.3)	2594 (30.0)	χ^2^_(1)_ = 950.58	< .001
F3 (*n*, %)	100,546 (27.4)	99,850 (27.9)	696 (8.1)	χ^2^_(1)_ = 1665.0	< .001
F4 (*n*, %)	16,934 (4.6)	16,552 (4.6)	382 (4.4)	χ^2^_(1)_ = 0.754	.385
F5 (*n*, %)	645 (0.2)	643 (0.2)	2 (< 0.1)	χ^2^_(1)_ = 11.75	.001
F6 (*n*, %)	15,554 (4.2)	15,111 (4.2)	443 (5.1)	χ^2^_(1)_ = 17.21	< .001
F7 (*n*, %)	2947 (0.8)	2923 (0.8)	24 (0.3)	χ^2^_(1)_ = 30.65	< .001
F8 (*n*, %)	252 (0.1)	246 (0.1)	6 (0.1)	χ^2^_(1)_ = 0.001	.978
F9 (*n*, %)	884 (0.2)	876 (0.2)	8 (0.1)	χ^2^_(1)_ = 8.10	.004
F1 + F2 (*n*, %)	24,660 (6.7%)	22,852 (6.4%)	1808 (20.9%)	χ^2^_(1)_ = 2848.37	< .001
F1 + F6 (*n*, %)	19,276 (5.3%)	18,334 (5.2%)	942 (10.9%)	χ^2^_(1)_ = 567.43	< .001
add. psych. diagn. (mean, SD)	1.41 (1.49)	1.4 (1.5)	2.03 (1.74)	*t*_(8914.27)_ = − 33.22	< .001
somatic diagn. (mean, SD)	2.29 (3.35)	2.32 (3.38)	1.18 (1.73)	*t*_(10299.27)_ = 58.62	< .001

F0 = organic, including symptomatic psychical disorders; F1 = substance use disorders; F2 = psychotic disorders; F3 = affective disorders; F4 = neurotic, stress-related and somatoform disorders; F5 = abnormal behavior with physical disorders and factors; F6 = personality disorders; F7 = reduction of intelligence; F8 = development disorders; F9 = behavioral and emotional disorders that begin in childhood and youth

**Table 2:** Treatment characteristics and group differences

	**Total** **(*N* = 366,767)**	**Non-homeless** **(*n* = 358,131)**	**Homeless** **(*n* = 8636)**	**Group comparison**	***p***
Treatment characteristics (mean, SD)					
Days of treatment	22.55 (28.16)	22.7 (27.96)	16.10 (35.08)	*t*_(8901.52)_ = 17.37	< .001
24-h cases (*n*, %)	37,744 (10.3)	36,195 (10.1)	1549 (17.9)	χ^2^_(1)_ = 560.0	< .001
Legal status at admission, PsychKG* (*n*, %)	39,959 (10.9)	38,427 (10.7)	1532 (17.7)	χ^2^_(3)_ = 430.80	< .001
Physical restraint (*n*, %)	6944 (1.9)	6711 (1.9)	233 (2.7)	χ^2^_(1)_ = 30.83	< .001
Number of days with restraint	0.1 (0.97)	0.10 (0.95)	0.14 (1.28)	*t*_(5414.45)_ = − 2.21	.027
Days with restraint per treatment day	0.01 (0.13)	0.01 (0.13)	0.02 (0.12)	*t*_(5268.70)_ = − 2.47	.013
Seclusion (*n*, %)	1379 (0.4)	1343 (0.4)	36 (0.4)	χ^2^_(1)_ = 0.39	.53
Number of days with seclusion	0.03 (0.63)	0.3 (0.64)	0.3 (0.51)	*t*_(5573.81)_ = − 0.63	.53
Days with seclusion per treatment day	0.0022 (0.08)	0.0022 (0.08)	0.0028 (0.05)	*t*_(5577.43)_ = − 0.86	.392
Number of somatic consultations	1.46 (0.93)	1.46 (0.93)	1.32 (0.72)	*t*_(56.40)_ = 1.46	.149
Somatic consultations per treatment day	0.10 (0.21)	0.10 (0.21)	0.09 (0.1)	*t*_(57.94)_ = 1.14	.258
Medical doctor units	3.4 (5.99)	3.42 (6.03)	2.21 (3.1)	*t*_(3350.88)_ = 20.41	< .001
Medical doctor units per treatment day	0.27 (0.83)	0.27 (0.84)	0.29 (0.44)	*t*_(3291.44)_ = − 1.80	.071
Staff nurse units	11.42 (24.63)	11.53 (24.74)	5.59 (16.73)	*t*_(4933.44)_ = 23.54	< .001
Staff nurse units per treatment day	0.76 (3.82)	0.76 (3.86)	0.49 (1.13)	*t*_(6652.98)_ = 14.85	< .001
Social worker units	3.74 (5.47)	3.74 (5.47)	3.58 (4.89)	*t*_(965.99)_ = 1.01	.366
Social worker units per treatment day	0.25 (0.77)	0.25 (0.77)	0.27 (0.52)	*t*_(980.92)_ = − 1.25	.211

^*^Legal status PsychKG = German Law on Help and Protective Measures for Mentally Ill Persons (Gesetz über Hilfen und Schutzmaßnahmen bei psychischen Krankheiten)

Table 2 shows that homeless patients had a shorter course of treatment and were significantly more often discharged within one day (24-hour cases). Involuntary admission and physical restraint occurred significantly more often in homeless patients. Regarding treatment resources, non-homeless patients obtained significantly more therapeutic units (one therapeutic unit = 25 minutes) from medical doctors and staff nurses. After adjusting for duration of treatment, this difference remained significant for staff nurse units but disappeared for medical doctor units (the trend even reversed). Groups did not differ in the quantity of social worker units obtained (Table 2).

PCA and logistic regression were chosen to determine the influential principal components on homelessness in psychiatric inpatients. The Kaiser-Meyer-Olkin measure of sampling adequacy was only .15, thus disabling factor analysis. Subsequently logistic regression was performed to determine predictive factors for dichotomous category membership (homeless vs. non-homeless). The model initially contained 19 independent variables, subdivided into patient-related (age, gender, psychiatric main discharge diagnosis, psychiatric comorbidity measured as number of secondary psychiatric diagnoses) and social–environmental variables at the hospital sites (number of inhabitants, homelessness, rate of migration and rate of poverty measured as number of households in the jobseeker subsistence system). The correlation between some predictor variables was high (*r* > .75), therefore rate of poverty measured as number of households in the jobseeker subsistence system was excluded as a variable and diagnoses were only considered if they had a frequency of at least 5% in the total sample. After this adjustment, multicollinearity was no longer a confounding factor in the analysis and correlations between predictor variables were low (*r* < .75). Goodness-of-fit was assessed using the Hosmer-Lemeshow test, indicating a poor model fit [χ^2^(8) = 97.23, *p* < .001].

The binomial logistic regression model was statistically significant [χ^2^(10) = 8074.45, *p* < .001] but Nagelkerke’s *R*^2^ was low at .110 and 97.6% of cases were already classified without further information. Thus, logistic regression was disabled as well.

## Discussion

Homelessness in countries with developed economies is a difficult and significant social issue, especially when it comes to disadvantaged groups such as psychiatric patients. Our retrospective data analysis of more than 360,000 inpatient psychiatric treatment cases conducted identified a rate of 2.4% homeless patients (with distinct regional differences), who 1) were mainly male (80%) and eight years younger; 2) more likely to have a psychosis and SUD diagnosis; 3) had more additional psychiatric and less somatic diagnoses; 4) had fewer treatment days and were more often discharged within one day; and 5) had more involuntary admissions and physical restraints compared to non-homeless patients.

The rates of homelessness among psychiatric patients in international studies differ considerably. In this context, the different methodological approaches have to be considered: there have been European [4, 24, 28, 30–32, 40, 43, 45] and north American studies [13, 25, 41] vs. analysis from low-income Afro-Asian countries [17, 33, 37, 44], evaluation of a complete cohort [4, 24, 30, 32, 43, 45] vs. a defined subgroup with distinct patient characteristics [13, 17, 25, 28, 31, 33, 37, 40, 41, 44], the inclusion of current homelessness vs. probability and history thereof [13, 30], a definition of literally homeless vs. no private home [40, 45]. A rate of 2,4% homelessness as observed in the present study, is a relatively low prevalence, but other European studies with a similar design (inclusion of all consecutive psychiatric admissions) also found single digit prevalences of 0.5-1.58% in Denmark (“one-year cumulative probability of first homelessness after discharge”, [30], 1.6% in Switzerland [24], and 3.8% in the UK (focus on ethnical differences in treatment, [4]. Previous Danish studies observed a rate of 6-8% [31, 32], and the recently published National Epidemiologic Survey on Alcohol and Related Conditions in the US observed a rate of 5% homelessness between the two waves of the survey (only participants with Alcohol and Related Conditions, [13]. The rate of 13% homelessness in the German WOHIN-study was based ona homeless definition including literally homeless, emergency shelter, homeless shelter, women’s refuge, refugee shelter, and improvised accommodation [40].

The sample of the present study is comprehensive and provides a large amount of representative data. The coverage of four years enabled us to identify an increasing trend of homelessness among inpatients in psychiatric hospitals in NRW from 2016 to 2019 (+14%). Whereas half of the total sample as well as of the not homeless subgroup was attributed to the LWL sector, only one third of homeless treatment cases were registered there. If calculated per 100.000 people or treatment cases respectively, the rates differed significantly between hospitals. Since sociodemographic data could only be analyzed with reference to the hospitals’ site (instead of the patients’ whereabouts), conclusions are limited. The three hospitals with the highest percentage of homeless treatment cases (Düsseldorf, Münster, Cologne) were not congruent with the cities with the largest homeless populations (Bedburg-Hau, Lengerich, Langenfeld), but partly represented the most populated NRW cities (Köln, Düsseldorf, Dortmund) with 360,000 to 1 million inhabitants. Moreover, Cologne and Düsseldorf have the largest train stations in NRW. Homeless patients therefore might not be registered inhabitants of those cities but use the train station as an easily accessible shelter. Homelessness seems often linked not only to the general social support system and the availability of affordable accommodation, but also to the arrangement of such public space [10]. The train station not only offers basic infrastructure (such as sanitary facilities and social services), qualifies as a meeting place to stay without allowance, and represents, albeit precariously, a way of participating in social life [23]. Nevertheless, the large differences in homelessness rates between hospitals cannot be explained satisfactorily. Possibly factors of the local social support system for the homeless and local socioeconomic factors may play a role. Homelessness is often assumed to be an urban phenomenon [8], because homeless people are more numerous, more geographically concentrated, and more visible in urban areas. Today, most of the world’s population is concentrated in urban centers. Urban homelessness, especially severe crowding, is the result of poverty and a lack of affordable housing and has risen disproportionately in areas with a shortage of affordable private rental housing and higher median rents [34]. But homelessness and housing exclusion are not just the prerogative of large cities [1].

With a focus on patient-immanent criteria, only one-fifth of our homeless sample was female. This result is congruent with other studies, which assessed male sex as a factor that most markedly differentiated homeless from domiciled patients [31, 32] and found about half (45%: [13] or even two thirds (70%: [24],75,8%: [40] of the homeless patients in psychiatry to be male. When interpreting this male predominance, it has to be taken into account that women more frequently tend to live “undercover” in hidden, insecure accommodation (such as in informal housing conditions, living temporarily with friends, relatives or a partner, living in women’s shelters). These women are, therefore, effectively “hidden” from view, are generally not counted as homeless and therefore are not shown in the statistics [6]. At the same time, this group of hidden homeless women is very vulnerable because of reported mental illness, being in local authority care, experience of domestic violence and other forms of abuse and with current drug or alcohol dependencies [6].

Homeless patients in the current study had a mean age of 39.27 years and were on average eight years younger than non-homeless patients. Lauber et al. observed a mean age of 34.4 years in their homeless population (2005) and homeless participants in the WOHIN study were also significantly younger, but it remained unclear whether this was due to the increasing rate of homelessness among mentally ill people or to the increasing mental morbidity among homeless people [39, 40]. The Danish studies considered young age as risk factor for homelessness in psychiatric patients [31] and found their homeless patients to be most often under 45 years of age [32]. Whether the younger age of homeless patients means that homelessness precedes the onset of mental illness (and is therefore not the precursor) or that their mental illness is so severe that they lose their housing within a short period cannot be answered with our current data (no information on first onset of the disorder vs. last date of accommodation). It may be possible that previously homeless persons of higher age have received more extensive psychosocial services and are more often accommodated in (long-term, assisted) housing facilities, whereas younger homeless persons have not received such psychosocial services yet or to a smaller extent and tend to be living more often as homeless and/or they may opt to do so.

In accordance with other studies [24, 31, 32, 39], homeless patients in the present study more often had a diagnosis of psychosis and SUD. Moreover, as observed in the Swiss cohort, the homeless as compared to other psychiatric inpatients had lower rates of affective disorders, but higher rates of having a dual diagnosis [24]. Such dual diagnosis might not only interfere with compliance and treatment itself, but SUD would even occur before onset of homelessness [39] and increase the risk thereof after discharge [30]. The fact that the number of additional psychiatric diagnoses in the homeless sample was significantly higher is compatible with a higher severity of the disorder.

On the other hand, the number of somatic comorbidities in the present study was significantly lower in the group of homeless patients, as seen in Switzerland [24]. Homeless patients did not receive more somatic consultations during the hospital treatment than patients with a fixed address. The significantly younger age of the homeless persons in our study may explain the low rates of somatic comorbidity. Still, this remains surprising because life on the street represents a major health burden, with evidence of accelerated ageing and high rates of acute and chronic infectious, circulatory, respiratory and musculoskeletal diseases [19, 22]. Again, access to sufficient but low-threshold and continuous medical care may be limited for the homeless and this may lead to underdiagnosis of somatic disorders. Or even if the homeless patients gain access as in our study, their shorter duration of treatment and as observed the high number of patients discharged within one day disables adequate physical examination with blood tests. Longer stays in somatic hospitals have been shown for homeless compared to house patients. But longer discharge delays were only significant in the absence of a psychiatric diagnosis [14]. The present data show this as well and are in accordance with the WOHIN study [40] and the analysis from Switzerland [24], where the length of hospital treatment was significantly shorter in homeless patients, who consecutively improved less [24]. The reasons for the shorter length of stay remain unclear and may include both patient- and service-related aspects. The higher number of 24-hour cases (discharged within one day) may be due to patient-related factors such as a diagnosis of substance use disorder, restlessness as a consequence of homelessness or individual pattern of coping. However, institutional factors also have to be considered and a long-term homeless patient without health insurance, with altered circadian rhythm and adjustment difficulties in strict institutional settings might be discharged earlier back onto the streets without adequate planning.

The present study found no difference between homeless and non-homeless patients in somatic consultations, medical doctor units or social worker units per treatment day and partly corresponds to Lauber et al., who did not observe disproportionate use of inpatient resources by homeless people (2005). Homeless psychiatric patients received or utilized even less staff nurse units per treatment day in this study. Homeless individuals are one socially disadvantaged population, sometimes experiencing stigma and discrimination in their psychosocial and health care seeking. Homeless persons in emergency situations might feel prejudged by clinicians as being drug-seekers. In a recent Canadian narrative inquiry, vulnerably housed individuals believed they received poor quality care or were even denied sufficient care for mental illness and addictions when clinicians became aware of their housing status [15]. Subsequently treatment can be further complicated by psychiatric comorbidities, missed appointments, and a lack of coverage for complementary therapies. Moreover, marginalizing behavior towards and inadequate address of patient’s concerns can negatively impact on their overall health-seeking behavior and engagement in the long run [36]. The fact that almost every other of the homeless patients in the present study (44,67%) was admitted outside regular working hours validates this assumption. Moreover, they had a higher rate of involuntary and therefore emergency admissions than non-homeless patients. This finding is in accordance with the Swiss cohort [24]. The predominant involuntary legal status in the homeless group of the present study was the German Law on Help and Protective Measures for Mentally Ill Persons (so-called PsychKG), which includes endangerment of others as potential reason for involuntary admission. The alternative involuntary legal status based on §1906 of the German Civil Law Code (so-called BtG) makes the persons’ welfare the most important priority and implies that a patient who refuses the admission to or tries to escape from hospital can be forced to receive clinical treatment if either a risk of endangerment of self exists or their health would be negatively affected. In order to issue an order for mental health care, the court requires not only a legal guardian’s application, but also a medical certificate of a treating doctor about the indication and necessity of such court-ordered treatment against the person’s will. In other words, to achieve such mandated treatment, some lead-time as well as targeted interdisciplinary cooperation is necessary. Not surprisingly, the rate of BtG admissions was low in the homeless group. Suicidal behavior and physical aggression against others often precede involuntary admission and behavioral aspects such as threats, agitation and physical aggression frequently lead to seclusion and use of restraint. There is no reason why a homeless patients per se would or should be more agitated or aggressive than domiciled patients. The later onset of treatment, a (comorbid) SUD, the feeling of discrimination through health care professionals and the loud, hectic and crowded emergency atmosphere with glaring lights and scarce privacy might nevertheless induce a certain agitation and confusion at the time of admission. Earlier research found that living situation was not associated with coercive measures [27]. Homeless patients in the present study still had more physical restraints, but the number of days with restraint as well as the rate of restraint events per treatment day did not differ between groups. This could be explained by the shorter duration of treatment in the homeless subgroup, but also by the need for restraint immediately following admission as mentioned above. In Contrast to Thomas et al. [41], who found forced medication to be more likely instituted in psychiatric inpatients who are in a homeless living situation [41], the groups in this study did not differ significantly regarding forced medication (data not shown).

The present study has limitations that need to be taken into account when considering its results. The use of inpatient routine data enabled us to investigate the housing situation of the largest group of German psychiatric inpatients published so far. At the same time, the retrospective nature of this study and the structure of the data only allow limited conclusions. Due to the nature of our data (inpatient data with categories such as “zip code/city” and “place of residency”), the imminent risk of becoming homeless or living in precarious living conditions could not be considered. We had no information on mental and psychosocial care before and after the inpatient stays of the included patients, which might have given important further information about the risk of homelessness. A potential methodological limitation is the lack of standardized assessment and documentation of the housing situation of psychiatric inpatients in the medical records analyzed. We were able to identify the homeless persons by categorizing the documented addresses and zip codes as homeless or non-homeless. However, because there is no standardized documentation field “homeless”, we may have slightly underestimated the percentage of homeless patients. Moreover, individual sociodemographic factors (such as income) and the local municipal support that may be available (and its use rate) were not available in our dataset. Such information would shed more light on the full extent of the problem of homelessness in mental healthcare inpatients and would enable specified analyses of the risk factors for homelessness among the mentally ill. Due to the structure of our data, we could only deduce assumptions from the sociodemographic data at the hospitals’ sites. This does not represent reality directly, since not all patients (especially not the homeless ones) derive from the hospitals’ catchment area, which furthermore can be quite large and inhomogeneous, especially in rural regions. The consideration of the sociodemographic profile of the patient’s whereabouts might produce more valid results regarding this aspect, but were not available for analysis.The influence of other important factors (e.g., family background, physical and cognitive development status, education, suicidality and aggressive behavior, treatment variables such as medication or past experiences regarding the healthcare system) on the housing status also could not be investigated because they are not documented in a standardized way or are currently documented in separate documentation systems that were not available in our dataset (such as medication data).

The presented retrospective part of our WohnLos-study considered housing situation prior to hospital admission and the nature of data did not allow analysis of the housing situation after psychiatric hospitalization discharge. Other work packages of this study explicitly dealt with the problem of homelessness as nonmedical reason for hospital discharge delays as well as with post-discharge pathways to subsequent housing (Ueberberg et al., in preparation).

The observed high regional variability of homelessness rates indicates that local factors (e.g., sociodemographic and socioeconomic factors) or the degree of development of the local municipal social support system may interact with individual risk factors at patient level. Further studies are warranted to include such potential local sociodemographic and social support factors with a view to identifying the strength of the relative contributions of these factors towards homelessness among inpatients with mental illness.

## Conclusions

Homelessness is an increasing social problem in Western European countries. In Germany, there has been an increase in homelessness in recent years. The coexistence of homelessness and mental disorders is evident. Mental illness is a risk factor for becoming homeless, but homelessness in itself also negatively affects mental health. Our study analyzed the problem of homelessness in a sample of 366,767 inpatient cases in 20 psychiatric hospitals in NRW, Germany, in the years 2016–2019. The routine data analysis established a 2.35% prevalence of homelessness, with vast differences of prevalence between hospitals (range: 0.2–6.3%).

Our analysis found that multiple individual sociodemographic and local factors are associated with the homelessness of psychiatric inpatients. In addition, clinical factors differ between homeless and non-homeless patients, pointing to more severe mental illness and treatment complications (e.g., more coercive measures) in homeless compared to non-homeless persons. Thus, homelessness of psychiatric inpatients can imply special challenges that need to be considered by healthcare providers and politicians, with the goal of optimizing mental and social care and the mental health outcomes of homeless persons.

## Data Availability

Aggregated data are available on request. Please contact I.S.H. as corresponding author.

## Abbreviations

DSM-IV: Diagnostic and Statistical Manual of Mental Disorders, Fourth Edition
ETHOS: European Typology of Homelessness and Housing Exclusion
ICD-10: International Classification of Diseases, Tenth Revision
LVR: Landschaftsverband Rheinland
LWL: Landschaftsverband Westfalen-Lippe
MAGS: Ministerium für Arbeit, Gesundheit und Soziales
NRW: North Rhine-Westphalia
PCA: principal component analysis
SUD: Substance Use disorder
